# Diffusion of a new intermediate product in a simple ‘classical‐Schumpeterian’ model

**DOI:** 10.1111/meca.12183

**Published:** 2017-07-28

**Authors:** David Haas

**Affiliations:** ^1^ University of Graz

## Abstract

This paper deals with the problem of new intermediate products within a simple model, where production is circular and goods enter into the production of other goods. It studies the process by which the new good is absorbed into the economy and the structural transformation that goes with it. By means of a long‐period method the forces of structural transformation are examined, in particular the shift of existing means of production towards the innovation and the mechanism of differential growth in terms of alternative techniques and their associated systems of production. We treat two important Schumpeterian topics: the question of technological unemployment and the problem of ‘forced saving’ and the related problem of an involuntary reduction of real consumption per capita. It is shown that both phenomena are potential by‐products of the transformation process.

## 1 INTRODUCTION

Economic development is a process in which new goods and methods of production get invented, some of which are adopted by the economic system, spread and gradually replace old ones. It is a process of ‘creative destruction’, as Schumpeter observed, that ultimately increases the material wealth of society. Innovations compel the economy to leave fully adjusted positions, or circular flows: they involve both the generation and destruction of economic variety (Schumpeter, 1934).

This paper deals with the introduction and diffusion of a new intermediate product in a simple model of production in which commodities are produced by means of commodities. It analyses the transition of the economy from a situation without the new intermediate product to one with it and sheds light on the conditions to be met for the process to take place and the impact it has on the system as a whole. The process will be studied from a classical‐cum‐Schumpeterian perspective.

The set‐up is the following: Prior to the innovation, only two commodities are being produced: a capital good that enters both in its own production and in the production of the second commodity, a pure consumption good. The capital good is thus the only ‘basic’ product in the system (cf. Sraffa, 1960). This structure gets disrupted by the invention, introduction and diffusion of a new intermediate product, which is produced by means of the capital good and is used in the production of the consumption good. Hence, as a result the formerly two‐commodity economy transforms into a three‐commodity one that employs what may be called a ‘more roundabout’ technique, using a concept of the Austrian economist Eugen von Böhm‐Bawerk.

The paper contributes to what is known as traverse analysis. Robert Solow once stated: ‘Traverse. That is the easiest part of skiing but the hardest part of economics’. The literature in the field under consideration arguably confirms this assessment. In order to render the problem manageable, we explore the traverse from what is known as the Hicks–Spaventa two‐good economy to the Lowe three‐good economy; for a comparison of the two models see Steedman (1998). Both models have been used to study aspects of the problem of the traverse within the given frameworks. In accordance with the literature, a traverse refers to the path triggered by a change in data, such as the set of available techniques, and the economy's response to such a change, that is, its movement from its old steady state to a new one. While the literature mentioned covers certain features of the traverse in each of the two models, a thorough analysis of the transition between the two economic structures has not been elaborated yet. Since many innovations involve the introduction of new intermediate products, the present study fills a gap. The paper pays particular attention to the process of construction and diffusion of the new technique.

The argument developed traces the time profiles of the movement of important economic variables and shows that different types or forms of innovation typically engender different effects. The first economist who was able to exemplify this fact in terms of a rudimentary traverse analysis couched in a famous numerical example was David Ricardo in the chapter ‘On machinery’ first published in the third edition of the *Principles* (see Kurz, 2010, 2015).

The approach chosen to investigate the problem of traverse between dimensionally different economic systems cross‐breeds Classical and Schumpeterian ideas along the lines proposed by Kurz (2008) and applies the specific variant of the long‐period method proposed by Metcalfe and Steedman (2013). This method addresses the long‐period forces and effects of economic development whose upshot is a sequential analysis regarding the adjustment path. The latter is characterized by the full utilization of capital goods and surplus labour. The analysis focuses attention on the conditions to be met by viable innovations, the role of capital transfers between industries and of differential rates of accumulation of rival systems of production (SoP's) along the traverse.

Two themes of Schumpeterian economics receive particular attention: the possibility of what is knowns as ‘technological unemployment’ and the concept of ‘forced saving’ and how these are determined and interrelated.

The main findings are the following: (a) Given Schumpeter's zero profit condition applied to the circular flow, a new intermediate product is economically viable if and only if it is labour‐saving. This need not be the case if the normal rate of profits is strictly positive. (b) The implementation of the new technique involves a time lag and requires that existing means of production are transferred between industries. Because of this, the introduction of a new intermediate product may under certain circumstances cause technological unemployment, or a reduction of real consumption per capita, or both. (c) If the greater surplus obtained by the new technique is invested, it gains economic weight through differential growth of rival SoP's. In this way, the structure of the economy gets transformed and employment grows. The effect of diffusion on real consumption depends on the ‘dynamism’ of the new industry with respect to the innovation surplus.

The paper is organized as follows: Section 2 introduces Schumpeter's notion of the circular flow and examines the question of economic viability of new intermediate products. Section 3 deals with the conditions and consequences of the construction and diffusion of the new technique which uses the new means of production. Section 4 concludes.

## 2 CIRCULAR FLOWS AND NEW INTERMEDIATE GOODS

We apply the analytic schema of Schumpeter (1934) and hence assume that the economy is in a ‘circular flow’, or stationary state, prior to the innovation and after having fully absorbed the novelty will be in such a state once again. A circular flow is a special case of a long‐period position characterized by (a) a cost‐minimizing system of production (SoP), (b) the absence of (extra) profits and (c) the absence of growth (Kurz, 2008). Metcalfe and Steedman (2013) emphasize that an important property of the circular flow is that in each industry a single method of production is used. This implies that any industry‐specific economic variety caused by the introduction and diffusion of new methods of production has been eliminated again via the competitive process. Therefore, in the new circular flow, as in the old one, the economy can only reproduce itself, but cannot evolve.

### 2.1 The ‘old’ circular flow

In the old circular flow two commodities are produced by means of the ‘old’ technique consisting of two methods of production, one for each commodity. The two methods are the following: producing one unit of good 1 (the capital good) requires *a*_11_ units of itself and *l*_1_ units of labour, while $a_{21}^{\left(o\right)}$ units of good 1 and $l_{2}^{\left(o\right)}$ units of labour produce one unit of good 2 (the consumption good). We assume that the system is strictly technologically viable, that is, $a_{11}<1$.

Since the rate of profits is taken to be zero in a circular flow, the ruling price system with the consumption good 2 as the numéraire is

(1) $$\begin{array}{c} p_{1}^{\left(o\right)}\;=\;p_{1}^{\left(o\right)}a_{11}+w^{\left(o\right)}l_{1},\\ 1\;=\;p_{1}^{\left(o\right)}a_{21}^{\left(o\right)}+w^{\left(o\right)}l_{2}^{\left(o\right)}, \end{array}$$

where $p_{1}^{\left(o\right)}$ denotes the relative price of good 1 and $w^{\left(o\right)}$ the real wage rate.

Because the rate of profits is zero, the labour theory of value holds (see, e.g., Kurz & Salvadori, 1995, p. 111). This means that relative prices are proportional to quantities of embodied labour:

(2) $$\begin{array}{c} p_{1}^{\left(o\right)}\;=\;v_{1}w^{\left(o\right)},\\ 1\;=\;v_{2}^{\left(o\right)}w^{\left(o\right)}, \end{array}$$

where *v*_1_ and $v_{2}^{\left(o\right)}$ are the quantities of labour embodied directly and indirectly in one unit of each of the two commodities.

As regards quantities, the input–output scheme of the initial circular flow is given by

(3) $$\begin{array}{c} x_{1}\;=\;a_{11}x_{1}+a_{21}^{\left(o\right)}x_{2}^{\left(o\right)},\\ x_{2}^{\left(o\right)}\;=\;w^{\left(o\right)}\left[l_{1}x_{1}+l_{2}^{\left(o\right)}x_{2}^{\left(o\right)}\right]. \end{array}$$

Here, production of the capital good 1, *x*_1_, equals the investments needed to reproduce exactly the same quantities that have been used up in the course of production; and production of the consumer good, $x_{2}^{\left(o\right)}$, equals total real wage payments since workers, by assumption, do not save. Consumption per unit of labour employed $c^{\left(o\right)}$ is thus equal to the real wage rate, and the uniform growth rate is zero. Before entering into a discussion of the process of transition from the old to the new circular flow we describe the properties of the latter.

### 2.2 The ‘new’ circular flow

In the new circular flow, after the economic system has become fully adjusted to the novelty, a ‘new’ technique is used, which now involves the production of three commodities. Whereas the method for commodity 1 is the same as in the old system, now two new methods occupy the stage. Firstly, there is a method by means of which one unit of commodity 3, the new intermediate product, is produced consuming productively *a*_31_ units of commodity 1 and employing *l*_3_ units of labour as inputs; secondly, there is a method producing the (unchanged) consumption good in a new way: $a_{23}^{\left(n\right)}$ units of commodity 3 and $l_{2}^{\left(n\right)}$ units of labour are needed to produce one unit of commodity 2.

Accordingly, normal prices corresponding to the new circular flow are given by

(4) $$\begin{array}{c} p_{1}^{\left(n\right)}\;=\;p_{1}^{\left(n\right)}a_{11}+w^{\left(n\right)}l_{1},\\ 1\;=\;p_{3}^{\left(n\right)}a_{23}^{\left(n\right)}+w^{\left(n\right)}l_{2}^{\left(n\right)},\\ p_{3}^{\left(n\right)}\;=\;p_{1}^{\left(n\right)}a_{31}+w^{\left(n\right)}l_{3}, \end{array}$$

where $p_{3}^{\left(n\right)}$ is the price of the intermediate product in terms of the consumption good, commodity 2.

Again, the labour theory of value holds in the new circular flow. That is to say,

(5) $$\begin{array}{c} p_{1}^{\left(n\right)}\;=\;v_{1}w^{\left(n\right)},\\ 1\;=\;v_{2}^{\left(n\right)}w^{\left(n\right)},\\ p_{3}^{\left(n\right)}\;=\;v_{3}w^{\left(n\right)}. \end{array}$$

The input–output scheme corresponding to the new circular flow is given by

(6) $$\begin{array}{c} x_{1}\;=\;a_{11}x_{1}+a_{31}x_{3},\\ x_{2}^{\left(n\right)}\;=\;w^{\left(n\right)}\left[l_{1}x_{1}+l_{2}^{\left(n\right)}x_{2}^{\left(n\right)}+l_{3}x_{3}\right],\\ x_{3}\;=\;a_{23}^{\left(n\right)}x_{2}^{\left(n\right)}. \end{array}$$

Here, $x_{2}^{\left(n\right)}$ is the quantity of commodity 2 produced by means of the new method (*n*) and *x*_3_ is the production of commodity 3.

Comparing the two circular flows, the following observations are close at hand. Firstly, in the old circular flow there is one *basic* product, the capital good, that enters the production of both products, and there is one *non‐basic* product that does not, the consumption good (see Sraffa, 1960). Actually, since product 2 enters the production of none of the products it is a *pure* consumption good. In the new circular flow, we have instead three products. The capital good is still the only basic product: it enters directly the production of the intermediate product and via the latter now indirectly the production of the consumption good, which is still a non‐basic. The intermediate product is a non‐basic, but one that is used exclusively as a means of production. Secondly, the new technique, if adopted, may be seen as reflecting a case of the larger productivity or *superiority of greater roundaboutness of production*. This concept played an important role in temporal capital theory advocated by ‘Austrian’ economists, especially Eugen von Böhm‐Bawerk (see Kurz & Salvadori, 1995, chaps. 6 and 14). The length of the overall production period of the consumption good has been increased by taking a route towards it via the intermediate product.

### 2.3 Economically viable new intermediate products

Now the question has to be addressed under which circumstances a more roundabout technique is cost‐minimizing. Put differently: Which types of new intermediate products are economically viable, that is, induce profit‐seeking agents to adopt them in the hope and expectation of exploiting their potential, and which will actually revolutionize the economic system via their diffusion?

The diffusion of a new intermediate product is technologically feasible if and only if two conditions are simultaneously met: (a) there must be a novel method that allows to produce the new intermediate product by means of already known and actually produced commodities—we call this the ‘producer method’; and (b) there must be a novel method using the new capital good to produce the known consumption good—we call this the ‘user method’. This double requirement cannot generally be assumed to be satisfied automatically. In case it is not, the proliferation of the new intermediate product may be delayed for a shorter or longer period of time or in the extreme even prevented forever.^1^

Since our main concern in this paper is with the problem of the economic viability of a new intermediate product, we for simplicity assume that both conditions are met and the respective methods are available. Based on Kurz (2008), who deals with the problem in regard to existing products and sets aside the introduction of entirely new ones, the new intermediate product is called economically viable if its production‐cum‐adoption does not incur extra costs at the prices ruling in a given situation. For the individual producers, this is so if

(7a) $$p_{3}\geq a_{31}p_{1}^{\left(o\right)}+w^{\left(o\right)}l_{3},$$

(7b) $$p_{1}^{\left(o\right)}a_{21}^{\left(o\right)}+w^{\left(o\right)}l_{2}^{\left(o\right)}\geq p_{3}a_{23}^{\left(n\right)}+w^{\left(o\right)}l_{2}^{\left(n\right)}.$$

The two inequalities show that what matters in this regard is the level of the price of the new commodity, *p*_3_: it must be high enough such that the producers of the new intermediate product obtain non‐negative profits (7a); at the same time it must be low enough such that the users of the new intermediate product do not incur extra costs (7b).

Let ${\underline{p}}_{3}$ ( ${\bar{p}}_{3}$) be the price at which the producer method (user method) obtains zero profits. Three cases are possible:

1. ‘Mere’ Invention: If ${\underline{p}}_{3}>{\bar{p}}_{3}$, there is no price at which both the production and the use would be profitable. In this case the new capital good cannot spread successfully, even if the diffusion happens to be feasible. This is the case of an invention that cannot become an innovation.
2. ‘Just viable’ Invention: If ${\underline{p}}_{3}={\bar{p}}_{3}$, the new capital good could be introduced without extra cost, but there would be no incentive to do so.
3. ‘Innovation’: If ${\underline{p}}_{3}<{\bar{p}}_{3}$, there is a whole range of prices of the new intermediate product at which both producing and using it is profitable. In this case the new intermediate product can be expected to diffuse—the invention will become an innovation.

Which case applies in any given situation can be shown to depend on the characteristic features of the two technical alternatives: Combining the two conditions (7a) and (7b) shows that the new intermediate product will gain economic weight, that is, become an innovation, if and only if it exhibits lower real unit costs (i.e., costs measured in terms of the consumption good), given the old (relative) price and the old real wage rate:

$${\underline{p}}_{3}<{\bar{p}}_{3}\Leftrightarrow \underset{=1}{\underset{︸}{p_{1}^{\left(o\right)}a_{21}^{\left(o\right)}+w^{\left(o\right)}l_{2}^{\left(o\right)}}}>p_{1}^{\left(o\right)}a_{31}a_{23}^{\left(n\right)}+w^{\left(o\right)}\left(a_{23}^{\left(n\right)}l_{3}+l_{2}^{\left(n\right)}\right).$$

Because commodity 2 is the numéraire and the uniform rate of profits is nil in the old circular flow, the ‘old’ real unit costs are equal to unity. Further, given the fact that the old relative prices are proportional to relative quantities of labour embodied (see equation system 2), it follows that the new intermediate product will be an innovation if and only if the new technique requires a smaller amount of direct and indirect labour to produce the consumption good than the old technique:

(8) $${\underline{p}}_{3}<{\bar{p}}_{3}\Leftrightarrow v_{2}^{\left(o\right)}>v_{2}^{\left(n\right)}.$$

Against the background of the theory of the *choice‐of‐technique* problem (see Kurz & Salvadori, 1995, chap. 5) this finding is not surprising: from condition (8) one can easily infer that the new technique is superior to the old one if and only if it is able to pay a higher real wage rate, given the rate of profits (or, alternatively, a higher rate of profits, given the real wage rate). This criterion extends also to our case, where certain goods are technique‐specific, as is shown by condition (8).

Notice that this condition depends, of course, on the Schumpeterian assumption entertained in the analysis that the rate of profits is nil in the old circular flow. As noted by Kurz (2008, p. 271), the ‘zero‐profits assumption […] implies that in order for an invention to become an innovation it *must* reduce labor costs’. Appendix A shows that in the presence of a positive rate of profits a labour‐saving bias is neither a sufficient nor a necessary condition for the new technique to qualify as an innovation.

## 3 THE TRAVERSE

We now turn to the process in the course of which the new technique gradually replaces the old one through the introduction of an economically viable new intermediate product and the corresponding new methods of producing and using it. We confine our analysis to the quantity side of the problem and consider only a particular type of traverse compatible with the long‐period method with a ‘classical’ flavour: we study the features of the adjustment path along which all produced commodities are continually fully utilized. Hence, the problems of idle products, inconsistent investment plans and of effective demand failures are set aside. The focus of our analysis are differential rates of accumulation of different types of capital goods.^2^

In order to get a clear picture of the role of real capital formation for the traverse under consideration, we assume that the expansion of productive capacity, or potential output, and that of actual output in effective demand coincide, but that surplus labour exists. That is, the classical concept of Say's Law, which does not include the labour market, but only the markets of produced commodities, is taken to hold along the path.^3^ This set of assumptions helps us to put into sharp relief certain long‐period forces of structural transformation.

A further assumption defines the sequence of events within a single production period, where, for simplicity, the production period is assumed to be uniform across all products. In addition, we assume that capital goods produced in period *t* are the means to produce goods in period *t* + 1, whereas consumption goods produced in period *t* are taken to be consumed in the same period *t*.

### 3.1 The construction phase of the new technique

We now turn to the adjustments through which the new technique emerges ‘from within’ the old one, that is, the old circular flow. In period −1 the new technique is in the making: the new intermediate product is being produced for the first time, but the new method using it is not yet being operated, because the means to do so are not yet available.

With regard to our simple case we address two issues: (a) the economy's ability to maintain its old circular flow level of employment; and (b) the possibility that the construction of the new intermediate product curtails real consumption per capita. Both issues play some role in Schumpeter's theory. The first one concerns the problem of *technological unemployment*, which he considered to be an unavoidable but temporary by‐product of the process of innovation and development (Hagemann, 2015, p. 128; see also Boianovsky & Trautwein, 2010). The second one relates to the idea of *forced saving*. In Schumpeter (1934, 1939), credit created by banks allows innovators to get hold of some of the amounts of the productive resources (labour and means of production) available in the circular flow in order to carry out their innovations. The increased monetary demand that meets with given amounts of productive resources is bound to lead to rising prices of some of these resources, that is, inflation. Since less of these resources are available to produce consumption goods, a reduction of real consumption per capita is considered to be also a likely, but temporary ‘real’ consequence of credit expansion. This is especially so, if existing means are fully utilized in the pre‐innovation situation and if the realization of the innovation involves what is called a ‘gestation period’, that is, a lag between the production of a new producer good and its use in the production of additional consumption goods; see Machlup (1943) on the concept of forced saving; see also Hagemann (2010) and Festré (2002).

In the following we elaborate on these ideas. In terms of our model, which is confined to the analysis to the ‘real’ side of the innovation process, it is shown that the two issues are closely intertwined and have a common cause.

#### 3.1.1 State of simple reproduction

We rewrite the input–output scheme of the economy corresponding to the old circular flow and indicate circular flow quantities by a bar on top of variables. The pre‐innovation situation is this:

(9) $${\bar{x}}_{1}\;=\;a_{11}{\bar{x}}_{1}+a_{21}^{\left(o\right)}{\bar{x}}_{2}^{\left(o\right)},{\bar{x}}_{2}^{\left(o\right)}\;=\;c^{\left(o\right)}\bar{L},$$

where consumption per capita $c^{\left(o\right)}$ equals the real wage rate $w^{\left(o\right)}$, and circular flow employment is $\bar{L}=l_{1}{\bar{x}}_{1}+l_{2}^{\left(o\right)}{\bar{x}}_{2}^{\left(o\right)}$. Up until period −2 the economy is assumed to be in this state of simple reproduction.

#### 3.1.2 Transfer of existing productive resources

In period −1 the new intermediate product, product 3, is produced for the first time. In order for innovators to be able do so, a transfer of existing means of production at the end of the preceding period is required. As Schumpeter (1934, p. 68) insisted in the case in which there in the initial situation the existing means of production happen to be fully employed, ‘the new combinations must draw the necessary means of production from some old combinations’; he added that ‘the carrying into effect of an innovation involves, not primarily an increase in existing factors of production, but the shifting of existing factors from old to new uses’ (Schumpeter, 1939, p. 110).^4^

At the end of period −2, when the economy still exhibits circular flow properties, the available quantity of good 1, ${\bar{x}}_{1}$, is divided among three uses: the new one, that is, the one associated with the production of the new intermediate product, and the two old ones, that is, those associated with the production of itself, the capital good and of the consumption good. Because existing amounts of the means are fully utilized and the possibility of attaining additional amounts (via imports or operating hitherto idle items) is ruled out, the quantity of it invested in the production of the new intermediate product is equal to the quantity withdrawn from existing uses:

$$\underset{‘\text{new}\;\text{investment}’}{\underset{︸}{a_{31}\Delta x_{3}}}=\underset{`‘\text{withdrawal}’}{\underset{︸}{-a_{11}\Delta x_{1}+\left(-1\right)a_{21}^{\left(o\right)}\Delta x_{2}^{\left(o\right)}.}}$$

Here, a $\Delta x_{i}$ indicates the difference between production of commodity *i* in period −1, which is $x_{i,\left(-1\right)}$, and in the old circular flow, which is ${\bar{x}}_{i}$.

Because there are two old uses, the start of production of the new commodity 3 is accompanied by either a decrease of production of commodity 1, or of commodity 2, or of both. Depending on the old use from which existing means are withdrawn, the change in the size of the two existing industries is given by

(10a) $$\Delta x_{1}=-\alpha \frac{a_{31}\Delta x_{3}}{a_{11}},$$

(10b) $$\Delta x_{2}^{\left(o\right)}=-\left(1-\alpha \right)\frac{a_{31}\Delta x_{3}}{a_{21}^{\left(o\right)}},$$

where α is the share of ‘new investment’ withdrawn from industry 1 and (1−α) is the share of ‘new investment’ withdrawn from industry 2. Since 0≤α≤1, at least one old industry must shrink.

#### 3.1.3 Employment effect

The shift of existing means of production from old uses towards the new one changes the size of existing industries, thereby changing the employment structure and, potentially, aggregate employment. The net employment effect, which is the sum of job destruction and job creation, is

$$\Delta L=\underset{\text{job}\;\text{destruction}}{\underset{︸}{l_{1}\Delta x_{1}+l_{2}^{\left(o\right)}\Delta x_{2}^{\left(o\right)}}}+\underset{\text{job}\;\text{creation}}{\underset{︸}{l_{3}\Delta x_{3}.}}$$

Taking into account Equations (10a) and (10b) shows that the net employment effect per unit of ‘new investment’ depends in general on the labour intensities of the three involved methods and on the ‘weights of withdrawal’, that is, on share α:

(11) $$\frac{\Delta L}{a_{31}\Delta x_{3}}=\alpha \left(\frac{l_{3}}{a_{31}}-\frac{l_{1}}{a_{11}}\right)+\left(1-\alpha \right)\left(\frac{l_{3}}{a_{31}}-\frac{l_{2}^{\left(o\right)}}{a_{21}^{\left(o\right)}}\right).$$

This equation states: (a) If the new producer method has the highest (lowest) labour intensity of all three operated methods, the net employment effect in period −1 is positive (negative). (b) If the labour intensity of the new producer method lies between the two ‘old’ labour intensities, the sign of the employment effect additionally depends on the weights of withdrawal: For a certain range of α, the employment effect will be positive, for another range it will be negative, and for a certain value of α it will be zero.

Overall, in a closed economy where capital is fully utilized, the construction of the new intermediate product can be expected to cause a change in employment, if this is effectuated through a shift of existing means of production between different uses. Only in certain special circumstances, for example, if all three methods have the same labour intensity, the net employment effect is zero. Technological unemployment, that is, a reduction of employment compared to the pre‐innovation circular flow situation, is likely to occur in the construction phase in cases in which the innovation withdraws resources particularly from relatively more labour intensive old uses.

#### 3.1.4 The problem of ‘forced saving’

We have shown that the shift of existing means towards the new use might both decrease the production of the consumption good and alter employment compared to the previous circular flow situation. If we insist on the full use also with respect to the consumption good (commodity 2), real consumption per capita may therefore be forced to adjust.

In our model, this is so because production of commodity 2, employment and real consumption per capita are related by

(12) $${\bar{x}}_{2}^{\left(o\right)}+\Delta x_{2}^{\left(o\right)}=\left(c^{\left(o\right)}+\Delta c\right)\left(\bar{L}+\Delta L\right),$$

for period −1. The LHS gives production and the RHS total real consumption demand; Δc denotes the change in real consumption per capita between period −1 and the circular flow.

From this equation, it follows that only in exceptional cases the old real consumption rate is exactly maintained in period −1, since this requires production of the consumption good and employment to change accordingly: $\Delta c=0\Leftrightarrow \Delta x_{2}^{\left(o\right)}=c^{\left(o\right)}\Delta L$. In general, the sign of Δc depends on the labour intensities of methods and on the weights of withdrawal. Consider the following three cases:

1. Increase of employment ( ΔL>0). Because existing means are shifted to the production of the new intermediate product, which provides the means needed to increase the production of the consumption good (using the new user method) not in this but in the subsequent period, the production of the consumption good cannot increase instantaneously. Due to this gestation lag, in cases in which the net employment effect is positive, real consumption per capita must fall irrespective of whether the consumption good industry shrinks or not due to the shift of means.
2. Withdrawal only from industry 2 (α = 0). If the means needed for the implementation of the innovation come exclusively from ‘old’ firms in the consumption good industry (α = 0), their output is bound to shrink ( $\Delta x_{2}^{\left(o\right)}<0$). In this case it can be shown that the reduction of production of the consumption good always outweighs a decrease in employment, if any (see Appendix B). As a result, real consumption per capita is reduced.
3. Withdrawal only from old industry 1 (α = 1). If the means needed for the implementation of the innovation come exclusively from firms in the capital good industry (α = 1), consumption good production remains at the old circular flow level ( $\Delta x_{2}^{\left(o\right)}=0$). In the case in which innovators happen to implement a producer method with a relatively smaller labour intensity compared to that of industry 1, employment falls ( ΔL<0), and real consumption per capita rises as a result ( Δc>0).

The first and the second case illustrate the two main conditions under which the construction of the new technique leads to a reduction of real consumption per capita. In the first case, this is so because the shift of means in favour of the production of the new means of production entails an increase of employment. In the second case, the reduction of real consumption per capita is caused by the shift of means from producing consumption goods to producing means of production, a phenomenon which may be called ‘forced accumulation’. The third case reflects the condition under which real consumption per capita is not reduced. This will happen if employment decreases and if the decrease of employment outweighs the reduction of consumption good production.

The second case is the one Schumpeter assumes in his ‘pure model’ of the capitalist process (Schumpeter, 1939, chap. IV). There he discusses the case of a new consumer good that requires a new capital good as an input and assumes that the ‘new investment’ is withdrawn exclusively from the ‘old’ firms producing the existing consumption good, that is, the case in which α = 0. He argues that ‘if there were only one single consumers’ good, less of it would be produced now than had been produced in the preceding state of equilibrium. Instead, more producers’ goods will be produced […] The output of consumers’ goods will fall in any case unless there is no period of gestation at all’. (Schumpeter, 1939, pp. 135–136) Since he assumes heterogeneous capital goods here, namely an ‘old’ one and a ‘new’ one, the statement on ‘more producers’ goods’ makes sense only if the stock of old capital does not shrink compared to the situation in the old circular flow, that is, if α = 0. Only in this case ‘forced accumulation’ in physical terms can be said to be a by‐product of the shift of means, enabled by credit creation.^5^ Note that in this case also the *value* of the capital stock (measured at old circular flow prices) clearly increases. However, if at least some resources are shifted from the old capital good industry towards the new one, the *value* of the capital stock need not always be relatively higher in the construction period.

#### 3.1.5 Discussion

We argued that both employment and real consumption per capita are likely to change if existing means of production are transferred from their previous uses to the building up of the new technique. We identified the labour intensities of the two old methods and of the new one and the weights of withdrawal as main determinants of what happens. In contrast to one‐good models (Metcalfe & Steedman, 2013; see also Haas, 2016), in multi‐good models such as the present one there are typically different types of old uses for existing means and, in the case of new capital goods, also gestation lags; this has been shown to be important for the effects during the construction phase, which do not only depend on the type of innovation, but also on the source(s) of the ‘new investment’ through which the innovation enters the economy.

Because initially the new investment can be expected to be relatively small, also the discussed effects will tend to be small; in our model they are nonetheless important since they affect the path the economy takes: If the initial shift of means reduces the size of industry 1, or more generally entails a de‐accumulation of the ‘old’ basic self‐reproducing system of the economy, also the amounts of means that can be used productively in total in the next period is smaller, meaning that whatever happens in the very beginning finds an ‘echo’ in subsequent periods.

We illustrated various cases in order to put the role of the weights of withdrawal into sharp relief, but we did not provide an argument on what determines them. To be sure, the specific type of long‐period method we applied here is not particularly well suited to deal with this problem, because the monetary aspects of innovations, the short‐run market price adjustments and expectations of existing firms are set aside, but can be expected to play an important role here.

Furthermore, our argument depends strongly on the implicit assumption that the shift of means from certain old uses to the new one does not provoke any further ‘second‐order shift’ at the end of period −2, namely one that re‐proportions the two existing industries in some way. For example, if producers in the consumption good industry were not assumed to be completely myopic but to expect that the innovation will cause a change in employment, and if they would be able to adjust their size accordingly, the change in real consumption per capita would be comparatively smaller. Overall, such ‘second‐order shifts’ would essentially imply that the withdrawal share α, which we here treated as exogenous, becomes endogenous. Appendix C deals with this issue in an indirect way by assuming that consumption per capita remains constant because a second‐order shift adjusts production of the consumption good to the change in employment. This exercise allows one to gain some insights into this problem.

### 3.2 Diffusion

Once the conditions for installing the new methods are met and the new intermediate product is available on the market, four methods are used in the economy: the established ‘old’ method in the basic capital good industry, the ‘new’ method that produces the new intermediate product, and two methods that produce the consumption good, where one is ‘old’ and one is ‘new’. The economy hence exhibits greater variety, which is a prerequisite for it to evolve through a process of differential growth. Through this process the economic weight of the new technique gradually increases and the economy structurally transforms itself.

#### 3.2.1 Two rival systems of production

Assume that all four methods are operated in period *t*. Because two production techniques are operated at the same time, the economy can be viewed as being composed of two systems of production (SoP's). The ‘old’ SoP (*o*) operates the old technique and requires two distinct activities, or components: component $1^{\left(o\right)}$ (re‐)produces commodity 1 for itself and for component $2^{\left(o\right)}$, which in turn produces commodity 2. The ‘new’ SoP (*n*) operates the new technique and consists of three distinct components: component $1^{\left(n\right)}$ (re‐)produces commodity 1 by means of the existing method, namely for itself and for component $3^{\left(n\right)}$, which produces the intermediate product; component $2^{\left(n\right)}$ produces commodity 2 using the intermediate product.

In period *t*, total production can thus be thought of as being the sum of outputs provided by the respective components of the two SoP's:

$$\begin{array}{c} \text{Industry}\;1:\;x_{1,t}\;=\;x_{1,t}^{\left(n\right)}+x_{1,t}^{\left(o\right)},\\ \text{Industry}\;2:\;x_{2,t}\;=\;x_{2,t}^{\left(n\right)}+x_{2,t}^{\left(o\right)},\\ \text{Industry}\;3:\;x_{3,t}\;=\;x_{3,t}^{\left(n\right)}. \end{array}$$

This decomposition of industry outputs (LHS) into the contributions of the two SoP's (RHS) is straightforward with respect to the new intermediate product 3, because only the new SoP produces it, and also with respect to product 2, because in this industry the two SoP's here operate different methods. The splitting up of industry 1, where both SoP's use the same method, is purely analytical.

The decomposition of the economy into the two SoP's will help us to identify a crucial driver of structural transformation: differential growth of rival SoP's.

#### 3.2.2 Innovation surplus and differential growth

We use a simple case to illustrate the mechanism of differential growth of alternative SoP's. To put this into sharp relief, two other adjustments are neglected, namely shifts of means from the old SoP to the new SoP and shifts of means among the components of a SoP. Further, we stick to the assumption that products are fully utilized.

For the transformation of the economy the new industry plays a key role, since its size limits the quantity of consumption goods that can be produced by means of the new ‘user method’, thereby determining the relative economic weight of the new technique. Its expansion therefore is central for the new SoP that replaces the old one.

The rate at which the new industry grows can be expected to be positively related to the extra profits obtained in this industry. Firstly, extra profits will motivate an early ‘swarm of imitators’ (Schumpeter, 1934) to shift additional means in the same way as the innovator has done in the construction phase. Secondly, retained extra profits provide innovators with the internal means to accumulate, an argument that is central to evolutionary models of competitive selection (Metcalfe, 1998, Montobbio, 2002). Concerning the latter, producers in the new industry can be taken to be in a favourable position because of two reasons: they can carry out their accumulation plans by getting the necessary inputs via paying a (marginally) higher price for them; and they are able to sell their product by charging a (marginally) lower price than the reservation price of potential customers. Their ‘marginal’ competitors cannot actually compete with them, because making nil profits at the old circular flow prices they would make losses in the new situation.

For what we want to show here, it is enough to assume that the new industry grows at some given and constant rate *g*_3_.^6^ Our decomposition implies that all other components of the new SoP (and the amount of labour it employs) must also grow at rate *g*_3_: Component $1^{\left(n\right)}$, which supplies component $3^{\left(n\right)}$ and itself with the basic capital good, must grow at rate *g*_3_ in order to be able to satisfy the growing requirement. And component $2^{\left(n\right)}$ must grow at rate *g*_3_ in order to be able to fully absorb the growing supply of the new intermediate product. Hence

(13) $$\frac{x_{1,t}^{\left(n\right)}}{x_{1,t-1}^{\left(n\right)}}=\frac{x_{2,t}^{\left(n\right)}}{x_{2,t-1}^{\left(n\right)}}=\frac{x_{3,t}^{\left(n\right)}}{x_{3,t-1}^{\left(n\right)}}=\frac{L_{t}^{\left(n\right)}}{L_{t-1}^{\left(n\right)}}=\left(1+g_{3}\right),$$

where $L_{t}^{\left(n\right)}=l_{1}x_{1,t}^{\left(n\right)}+l_{2}^{\left(n\right)}x_{2,t}^{\left(n\right)}+l_{3}x_{3,t}^{\left(n\right)}$ is total employment of the new SoP in period *t*. Furthermore, it must hold that

(14) $$\begin{array}{c} x_{1,t-1}^{\left(n\right)}\;=\;\left(1+g_{3}\right)\left(a_{11}x_{1,t-1}^{\left(n\right)}+a_{31}x_{3,t-1}^{\left(n\right)}\right),\\ x_{3,t-1}^{\left(n\right)}\;=\;a_{23}^{\left(n\right)}x_{2,t}^{\left(n\right)}, \end{array}$$

so that the new SoP is able to grow in a self‐sustained way. Notice that the relative size of the three new components depends both on capital coefficients and on the dynamism of the new industry. Since the new SoP grows at a uniform rate (Equation (13)) and is ‘well‐proportioned’ (Equation (14)), we can rely on the well‐known growth‐consumption curve to describe it. This relationship tells us that the higher the growth rate of the new SoP, which is *g*_3_, the lower is the quantity of consumption goods per unit of labour the new system employs, which is given by $x_{2,t}^{\left(n\right)}/L_{t}^{\left(n\right)}$.^7^

Similarly, the old SoP can be expected to return to its pre‐innovation ‘normal’ and well‐proportioned state of re‐production after the end of the construction phase, although at a slightly smaller scale due to the innovation withdrawal, because we set aside continuous shifts of means between the two SoP's in the simple case under consideration. The old SoP will thus exhibit zero growth and the old rate of real consumption per capita, which is $x_{2,t}^{\left(o\right)}/L_{t}^{\left(o\right)}=c^{\left(o\right)}=w^{\left(o\right)}$.

**Figure 1:**
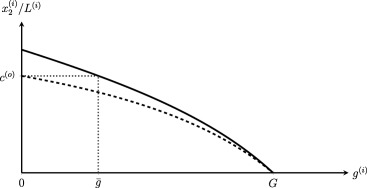
Illustration of consumption‐growth curves for the old system of production (dashed line) and the new system of production (solid line).

Figure 1 illustrates the consumption‐growth curve for a pair of old and new SoP's.^8^ Both SoP's have the same maximum growth rate *G*, since they utilize the same method in the production of the basic capital good. But because the new technique is an innovation (see Section 2.3), the new SoP produces a greater *surplus*, since for $x_{2,t}^{\left(n\right)}/L_{t}^{\left(n\right)}=x_{2,t}^{\left(o\right)}/L_{t}^{\left(o\right)}=c^{\left(o\right)}$ it can grow faster than the old one.

The difference in surplus rates can be expected to trigger a process of differential ‘normal’ growth of the two rival SoP's. The consequences of this process depend to a large extent on how the innovation surplus is spent. In the following it is assumed that the growth rate of the new SoP *g*_3_ is strictly positive, since otherwise the innovation cannot diffuse.

#### 3.2.3 Structural changes

One consequence of differential ‘normal’ growth of the two rival SoP's is structural change, both within and between industries. Here we only touch upon this issue in order to prepare the discussion of employment and real consumption dynamics.

As regards structural change within industries, the type of innovation considered here causes a variety of methods only in the consumption good industry; the latter undergoes a process of transformation in which the relative economic weights of the two available methods, the old method and the new user method, change.

Let $q_{2,t}=x_{2,t}^{\left(n\right)}/x_{2,t}$ be the output share of the new user method in period *t*, in which $x_{2,t}=x_{2,t}^{\left(n\right)}+x_{2,t}^{\left(o\right)}$ is total output of product 2. This output share also measures the economic weight of the new intermediate product and therefore its level of diffusion at any point in time. In our simple case, the rate at which the output share changes depends on the ‘dynamism’ of the new industry:

$$\frac{q_{2,t}-q_{2,t-1}}{q_{2,t-1}}=\frac{g_{3}-g_{2,t-1}}{1+g_{2,t-1}}=\left(1-q_{2,t-1}\right)\frac{g_{3}}{1+q_{2,t-1}g_{3}},$$

where $g_{2,t-1}$ is the aggregate growth rate of industry 2, given by the average rate of growth of the two SoP's with output shares as weights. It adapts from period to period in response to changing weights. This equation illustrates that the mechanism of differential growth in terms of the two SoP's generates the well‐known S‐shaped diffusion pattern—a stylized fact of diffusion research. But differential growth does not generate a simple logistic curve, because the total quantity of product 2 evolves at a non‐constant rate (Metcalfe & Steedman, 2013). Note also that the logistic pattern, a typical result of evolutionary diffusion models, depends crucially on the availability of surplus labour (Haas, 2016).

Structural change of industries, that is a change in the relative composition of industries, is another consequence of differential growth of rival SoP's. Industry *i* grows at the average rate at which the two components $i^{\left(n\right)}$ and $i^{\left(o\right)}$ grow with the respective industry output shares as weights. Since the development of these weights differs between industries, it is impossible for industries to grow uniformly along the traverse. We rather have differential growth rates across industries and time.

Especially in the early phase of the traverse, where the economic weight of the old SoP is still substantial, industry growth rates differ widely: The relative weight of the new SoP in the new industry 3 equals unity throughout, meaning that the growth rate of this industry is *g*_3_ along the whole traverse. Since initially the relative contribution of the new SoP in industries 1 and 2 is tiny, the two old industries grow relatively slowly in the beginning of the traverse. Later on, with increasing weights of the respective new components, the growth rates of the two old industries rise. And only in the limit, when their growth rates converge towards *g*_3_, they will fully catch up, indicating the completion of the traverse process. Hence, for a long time the new industry outpaces the rest of the economy; its dynamism only gradually propagates into those old industries to which the new SoP contributes.

#### 3.2.4 Employment growth

Total employment in the economy is given by $L_{t}=L_{t}^{\left(n\right)}+L_{t}^{\left(o\right)}$. Its growth rate is the weighted average of the growth rates of the two SoP's with their employment shares as weights. In our simple case of differential ‘normal’ growth of two rival SoP's, where the old system stagnates and the new one grows at rate *g*_3_, the employment growth rate is given by

(15) $$\frac{L_{t}-L_{t-1}}{L_{t-1}}=g_{3}\frac{L_{t-1}^{\left(n\right)}}{L_{t-1}},$$

where $L_{t-1}^{\left(n\right)}/L_{t-1}$ is the employment share of the new SoP in period *t* – 1. This growth rate is always positive and as long as there is surplus labour it is driven by the growth of the new SoP.^9^

Given our assumptions, technological unemployment therefore is only a temporary problem at the very beginning of the traverse that gets resolved later through surging surplus investments. This result can be said to partly support Schumpeter's opinion, that ‘the capitalist process has always absorbed, *at increasing real wage rates*, not only the unemployment it generated but also the increasing population’ (Schumpeter (1946) 1951, p. 200; cited in Boianovsky & Trautwein, 2010, p. 243; italics in the original).

#### 3.2.5 The problem of ‘forced accumulation’

We now study the consequences of differential growth on real consumption per capita by comparing paths that differ as regards the utilization of the innovation surplus.

In contrast to the situation in the construction period, during the diffusion phase both SoP's produce consumption goods. In period *t*, consumption per capita *c_t_*, assumed to be the same for all workers, is therefore determined by

$$x_{2,t}^{\left(n\right)}+x_{2,t}^{\left(o\right)}=c_{t}\left(L_{t}^{\left(n\right)}+L_{t}^{\left(o\right)}\right).$$

The LHS is total production of consumption goods and the RHS is total real consumption demand for it, that is real consumption per capita *c_t_* times total employment. By assumption, real consumption per capita adjusts in such a way that the consumption good market clears. We can re‐write this equation as:

(16) $$c_{t}=\frac{x_{2,t}^{\left(o\right)}}{L_{t}^{\left(o\right)}}\frac{L_{t}^{\left(o\right)}}{L_{t}}+\frac{x_{2,t}^{\left(n\right)}}{L_{t}^{\left(n\right)}}\frac{L_{t}^{\left(n\right)}}{L_{t}}=c^{\left(o\right)}+\frac{L_{t}^{\left(n\right)}}{L_{t}}\left(c^{\left(n\right)}-c^{\left(o\right)}\right).$$

It shows that real consumption per capita is the weighted average rate of real consumption of the two SoP's, again with employment shares as weights.^10^ Referring back to Figure 1, three cases are possible:

1. $g_{3}<\bar{g},\;c^{\left(n\right)}>c^{\left(o\right)}$. The new SoP does not fully exhaust its innovation surplus for the purpose of accumulation, with the effect that average real consumption increases over time.
2. $g_{3}=\bar{g},\;c^{\left(n\right)}=c^{\left(o\right)}$. Real consumption per capita remains at the old circular flow level, because at this specific growth rate the new SoP spends exactly its innovation surplus on growth.
3. $g_{3}>\bar{g},\;c^{\left(n\right)}<c^{\left(o\right)}$. The new SoP spends more than its innovation surplus on growth, which implies that real consumption per capita is bound to fall.

The first case shows that an under‐utilization of the innovation surplus for accumulation purposes impacts upon the well‐known static consumption‐growth trade‐off at the aggregate level: Differential growth increases the weight of the new SoP and hence shifts the aggregate consumption‐growth curve gradually outwards with the effect that both the economy's growth rate and real consumption per capita continually increase during the traverse. The drawback of comparatively low growth of the new SoP is, of course, that also the speed of diffusion and thus the rate of technical change is comparatively low. In the second case, in which the whole innovation surplus is spent on accumulation, real consumption per capita stagnates at the pre‐innovation level along the traverse but diffusion is completed more rapidly. Only in the third case a continual increase of the economy's growth rate is accompanied by a continual decrease of real consumption per capita. What may be termed ‘forced accumulation’ is caused by an overly dynamic new industry which pushes the growth rate of the new SoP beyond the innovation surplus it yields. This is likely to happen if the extra profits of the new technique do not percolate evenly into the economy but amass in the new industry which produces the new capital good, thereby inducing its fast growth.

Hence, not only the construction but also the diffusion of a new technique may, under certain circumstances, cause a reduction of real consumption per capita. Therefore, the problem of ‘forced saving’ may not be only a temporary problem, but may exist for a longer period of time, depending on how the innovation's extra profits are distributed across old and new methods.

## 4 CONCLUSIONS

The paper discusses the problem of the arrival and diffusion of a new intermediate product within a simple multi‐product economy. It highlights important ‘Schumpeterian’ features of the evolving economy in which the new technique, which brings the new good into the system, is first constructed and then diffused. We apply a specific version of the long‐period method, which leads us to a sequential study of the adjustment path along which two alternative techniques are used, goods are fully utilized throughout and there is surplus labour to accommodate the process. This helps us to clarify the role of capital re‐allocation and of differential growth of distinct SoP's during the transformation process.

The main findings are the following: (a) Innovations typically both increase and then decrease variety, by channelling new methods of production and the corresponding new intermediate products or capital goods, into the system and then by superseding old methods and means. (b) The particular type of innovation discussed in the paper has ‘Austrian’ features and reflects a case of the superiority of more roundabout methods of production. (c) Assuming with Schumpeter that the original circular flow is characterized by a zero rate of profits, the new technique is superior to the old one if the consumption good, which is taken to be the same between techniques, can be produced with a smaller amount of direct and indirect labour. (d) In the case of a positive rate of profits this condition does not apply (see Appendix A). (e) The process of the diffusion of the new technique and its gradual replacement of the old one depends on a number of factors. These include especially the following ones: the kind of innovation under consideration and how it compares technically with the old one; the amount of surplus, or profits, that can be generated by introducing the new technique, and the allocation of the surplus among the various industries; the amount of the surplus that is used productively, that is, invested in order to propel the diffusion of the new and spur economic growth; whether whatever is being produced is getting utilized (which is assumed in the analysis) and thus whether problems of effective demand can be put on one side; the environment into which the innovation is born, especially whether the growth of the system meets with an elastic supply of labour. (f) In case the amount of labour is elastic with regard to the needs of accumulation, while there may be ‘technological unemployment’, it will be limited to the early phase of the traverse process. (g) The introduction and diffusion of a new technology typically leads to differential growth rates among industries, whose spread is initially big and then gradually gets smaller. (h) A stylized fact of diffusion research is confirmed: the sigmoid pattern of the diffusion process, but differential growth does not result in a simple logistic curve.

Overall, the theoretical exercise provides a first approximation to the problem of evolutionary growth in the presence of production links among the various distinct activities, exemplified by means of a specific, more roundabout type of innovation.

We may conclude by pointing out that certain findings, such as those related to the gestation lag, depend on the type of innovation contemplated. Yet, the simple analytic schema may well be applied to study various other case. Such an extension could help to clarify how different types of innovations cause different problems along the path and entail different forms of structural transformation. A multi‐product framework, which takes into account produced means of production, offers the potential for a rich typology of innovations, forms of creative destruction and of obsolescence and degrees of disruption. To contrast our case, in which the new technique is accompanied by a new intermediate product, one can imagine the opposite case of a ‘less roundabout technique’ whose adoption renders an existing intermediate product obsolete.

## Figures and Tables

**Figure 2:**
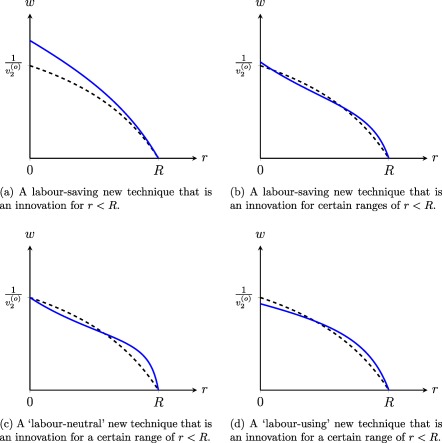
Wage‐profit curves for different types of new techniques. Wage‐profit curves of new techniques are graphed as solid lines, the wage‐profit curve of the old technique as a dashed curve. The numerical values of the old technique are $\left(a_{11},l_{1}\right)=\left(0.50,1.00\right)$ and $(a_{21}^{\left(o\right)},l_{2}^{\left(o\right)})=\left(0.60,4.00\right)$. As regards the new techniques, for (a), $(a_{31},l_{3})=\left(0.45,1.75\right)$ and $(a_{23}^{\left(n\right)},l_{2}^{\left(n\right)})=\left(0.60,2.50\right)$; for (b), $(a_{31},l_{3})=\left(0.10,2.30\right)$ and $(a_{23}^{\left(n\right)},l_{2}^{\left(n\right)})=\left(1.60,1.00\right)$; for (c), $(a_{31},l_{3})=\left(0.10,5.00\right)$ and $(a_{23}^{\left(n\right)},l_{2}^{\left(n\right)})=\left(0.85,0.78\right)$; for (c), $(a_{31},l_{3})=\left(0.25,1.75\right)$ and $(a_{23}^{\left(n\right)},l_{2}^{\left(n\right)})=\left(0.70,4.00\right)$. [Colour figure can be viewed at http://wileyonlinelibrary.com]

## Footnotes

1 There may be cases in which a new means of production is designed and put up for sale, initially only in the hope and expectation that some feasible and profitable application of it will be developed by others. Such *complementary innovations* are said to play a particularly important role for the development and diffusion of what are called general purpose technologies (GPTs); on GPTs see Bresnahan and Trajtenberg (1995) and Bresnahan (2010). See Rainer and Strohmaier (2014) and Strohmaier and Rainer (2016) for theoretical and empirical studies of GPT diffusion within a Sraffa–Leontief framework.

2 This method is used inter alia by Metcalfe (2007) in the context of a single industry model and Metcalfe and Steedman (2013) within a one‐commodity growth model. For long‐period models of differential but ‘normal’ growth, see also Metcalfe (1998).

3 This is a crucial assumption because the question of whether surplus labour exists or not changes the process by which new methods are absorbed into the system via the investment process (Metcalfe and Steedman, 2013); see also Haas (2016).

4 In a footnote of *Business Cycles* he considered this to be important for his theory of economic development, in particular because ‘in the traditional model it was increase in factors, rather than the shifting of factors, that was made the chief vehicle of economic progress. But essential phenomena of the cyclical process depend on that shifting of factors’. (Schumpeter, 1939, p. 110)

5 It is interesting to note that Schumpeter (1939, chap. IV) does not refer to the idea of forced saving explicitly and also leaves open the question of a reduction of real consumption per capita here (on this see Machlup, 1943, pp. 27–28). But he clearly indicates the possibility of a reduction by stating that ‘[i]t should be observed, however, that demand in terms of money for consumers’ goods has not decreased. On the contrary, it has increased’ (Schumpeter, 1939, pp. 135–136).

6 Implicitly this means that we limit ourselves to the profit‐propelled accumulation of existing producers, that is, the innovators plus, perhaps, the early swarm of imitators, and do not take into account continual imitation since otherwise the growth rate of the new industry could not be taken to remain constant over time.

7 Since two SoP's exist, this ratio is not equal to average consumption per capita in general (see below).

8 The numerical values are those of the first example discussed in Appendix A.

9 The employment effect of the diffusion of a new technique is unambiguously positive here, because we do not take into account that means are shifted between or within the two SoP's. As we have shown in our study of the construction period, such shifts can cause a net destruction of jobs, depending on the sign of the labour intensity differentials of methods (components) involved. Additionally, the direction of shifts of means, that is, towards producing more capital goods or towards producing more consumption goods, finds an ‘echo’ in subsequent periods. For example, a shift of means towards producing more capital goods may initially cause the net destruction of jobs, but may increase the stock of means (and hence employment) in the following period.

10 More precisely, $c^{\left(n\right)}$ is the quantity of consumption goods produced by the new SoP per hour worked in the new SoP as a whole. Since there are two SoP's it should be interpreted not as actual or average real consumption per hour worked in the economy.

## APPENDIX A ECONOMICALLY VIABLE NEW TECHNIQUES

We show that condition (8), according to which a new technique will be adopted, that is, become an innovation, if and only if it directly or indirectly saves labour in the production of the consumption good, does not carry over from a situation with a zero rate of profits to one with a positive rate: in the latter case, the condition is neither sufficient nor necessary.

To this end we illustrate the wage‐profit curves of the new and the old technique. In the case under consideration in the above, the two techniques differ with respect to the method of production for the non‐basic and pure consumption good 2, the latter being the same in both SoP's. Bharadwaj (1970, pp. 416–417) has shown that for two adjacent techniques using different methods in the production of only one of the common non‐basic commodities, the maximum number of switches is given by the number of different basic and non‐basic commodities that are used productively by at least one of the alternative methods of production for the non‐basic good mentioned. In our case this means that the new technique (*n*) and the old technique (*o*) have no more than two switches in the range 0≤r<R, where *r* gives the uniform rate of profits and *R* is the maximum rate of profits. Since the two techniques operate the same method of production for the only and common basic commodity, product 1, the maximum rate of profits is the same for both techniques: $R=R^{\left(n\right)}=R^{\left(o\right)}=\left(1-a_{11}\right)/a_{11}$. Therefore, the two techniques have an additional switch at *r* = *R*.

Since the two techniques use different methods to produce the common non‐basic commodity, that is, the consumption good 2, they can have different maximum wage rates, which are given by the respective inverse expressions of the quantities of labour embodied in one unit of the consumption good. We shall call a new technique ‘labour‐saving’ if $v_{2}^{\left(n\right)}<v_{2}^{\left(o\right)}$, ‘labour‐using’ if $v_{2}^{\left(n\right)}>v_{2}^{\left(o\right)}$, and ‘labour‐neutral’ if $v_{2}^{\left(n\right)}=v_{2}^{\left(o\right)}$.

Figure 2a illustrates the wage‐profit curve of a ‘labour‐saving’ new technique which has only one switch, namely at *r* = *R*. Hence, the new technique is an innovation for every 0≤r<R. Figure 2b also shows a ‘labour‐saving’ new technique, but one that exhibits two switches with the old technique. Because the new technique is an innovation only for those ranges of *r*, where its wage‐profit curve lies above that of the old technique, saving embodied labour, that is, $v_{2}^{\left(n\right)}<v_{2}^{\left(o\right)}$, is not a *sufficient* condition for the new technique to be economically viable in general.

Figure 2c shows a ‘labour‐neutral’ new technique which has three switches, including one at *r* = 0 since $v_{2}^{\left(n\right)}=v_{2}^{\left(o\right)}$. Nonetheless, it is an innovation if *r* lies above a certain level (but below *R*); this example indicates that the relative curvature of the two wage‐profit curves plays a role in certain circumstances. As Figure 2d illustrates, also a ‘labour‐using’ new technique can be an innovation for a certain range of *r*, if there is a switch at some 0<r<R. These two examples show that $v_{2}^{\left(n\right)}<v_{2}^{\left(o\right)}$ is not a *necessary* condition for the new technique to be strictly economically viable in general.

## APPENDIX B CONSTRUCTION: ‘FORCED SAVING’

Here we consider the question of ‘forced saving’, which we define as a situation in which means of production are shifted towards the construction of a new capital good with the effect of a falling consumption per capita. It is shown that if the withdrawal of resources reduces *only* the output of the consumption good industry, real consumption per capita is lower in the construction period (period −1) compared to the previous circular flow level.

Assume that in period −1 entrepreneurs, needing resources to carry out their innovations, manage to withdraw resources devoted to the production of the consumption good in the old circular flow but none from the resources devoted to the production of the basic capital good, that is, α = 0. From this shift of means from producing consumption goods towards producing the new intermediate product two consequences follow: According to Equation (10b) the change in production of commodity 2 is

(B1) $$\Delta x_{2}^{\left(o\right)}=-\frac{a_{31}}{a_{21}^{\left(o\right)}}\Delta x_{3},$$

and, according to Equation (11), the change in employment is determined by

(B2) $$\Delta L=\underset{\text{job}\;\text{destruction}}{\underset{︸}{l_{2}^{\left(o\right)}\Delta x_{2}^{\left(o\right)}}}+\underset{\text{job}\;\text{creation}}{\underset{︸}{l_{3}\Delta x_{3}}}=\left(\frac{l_{3}}{a_{31}}-\frac{l_{2}^{\left(o\right)}}{a_{21}^{\left(o\right)}}\right)a_{31}\Delta x_{3}.$$

Production of the consumption good therefore shrinks, whereas the sign of the net employment effect depends on the labour intensities of the two involved methods.

From Equation (12) it follows that real consumption per capita falls, that is, Δc<0 if and only if

$$\Delta x_{2}^{\left(o\right)}<c^{\left(o\right)}\Delta L.$$

By using Equations (B1) and (B2), we find that for the case of α = 0, real consumption per had must fall regardless of the sign and magnitude of the labour intensity differential:

$$\Delta c<0\Leftrightarrow \frac{\Delta L}{\Delta x_{2}}<\frac{1}{c^{\left(o\right)}}\Leftrightarrow -\frac{l_{3}}{a_{31}}<v_{1}.$$

This shows that if all the means needed by innovators are withdrawn from resources of existing producers of the consumption good industry, consumption per capita must fall, since the latter inequality condition is always met for non‐negative production coefficients. Only in the special case, in which the new industry operates a fully automated producer method, that is, $l_{3}=0$, consumption per capita would remain at the old circular flow level.

## APPENDIX C CONSTRUCTION PHASE: ‘SECOND‐ORDER SHIFTS’

Assume that innovators withdraw resources for ‘new investment’ from some existing use which provokes in turn an additional ‘second‐order’ shift that happens to keep real consumption per capita at its old circular flow level. That is, the production of good 2 is endogenously determined by the initial shift towards the new intermediate product and the change in employment caused thereby.

The assumptions of full utilization and of a constant real consumption per capita ( $\Delta c^{\left(o\right)}=0$) in period −1 requires that

(C1) $$a_{31}\Delta x_{3}\;=\;-a_{11}\Delta x_{1}-a_{21}^{\left(o\right)}\Delta x_{2}^{\left(o\right)},\Delta x_{2}^{\left(o\right)}\;=\;c^{\left(o\right)}\Delta L,$$

where $\Delta L=l_{1}\Delta x_{1}+l_{2}^{\left(o\right)}\Delta x_{2}^{\left(o\right)}+l_{3}\Delta x_{3}$. A constant real consumption per capita means that the change in production of good 2, that is, $\Delta x_{2}^{\left(o\right)}$ is now determined by the change in employment, which is ΔL, and not by some exogenous weights of withdrawal as above. Rather, if the shift towards the new good increases employment, production is supposed to increase accordingly by an additional shift of existing means from the old industry 1 to the old industry 2.

In this case, the change in the size of the two old industries is given by

(C2) $$\Delta x_{1}\;=\;-\underset{\;=\;\hat{\alpha }}{\underset{︸}{\left(\frac{v_{3}a_{11}}{v_{1}a_{31}}\right)}}\frac{a_{31}\Delta x_{3}}{a_{11}},\Delta x_{2}^{\left(o\right)}\;=\;c^{\left(o\right)}\Delta L\;=\;\underset{\;=\;-\left(1-\hat{\alpha }\right)}{\underset{︸}{\frac{a_{11}}{v_{1}}\left(\frac{l_{3}}{a_{31}}-\frac{l_{1}}{a_{11}}\right)}}\frac{a_{31}\Delta x_{3}}{a_{21}}.$$

Compared with the case above (see Equations (10a) and (10b)), the withdrawal share $\hat{\alpha }$ is now endogenously determined; it depends on the production coefficients of the methods that produce the two capital goods: (a) If the new producer method has a higher labour intensity compared to the method of industry 1, employment increases and production of good 2 increases correspondingly. The additional means to do so necessarily come from industry 1, which therefore shrinks faster than compared to the above case, such that in this case $\hat{\alpha }>1$. (b) If the shift of resources decreases employment (which is the case if the new producer method has a lower labour intensity compared to the method of industry 1), industry 2 shrinks, that is, $\hat{\alpha }<1$, and means of production are shifted from old industry 2 to old industry 1. This second‐order shift has the effect of indirectly decreasing both old industries because of the construction of the new product.
